# Not every knee tumour is a ganglion - retrospective analysis of benign and malign tumour entities around the knee

**DOI:** 10.1007/s00402-024-05401-7

**Published:** 2024-06-21

**Authors:** Peter Behrendt, T Grunow, Frosch K-H, M Krause, H Fahlbusch, M Priemel

**Affiliations:** 1 Department of Orthopedic and Trauma Surgery, University Medical Center Schleswig-Holstein, Campus Kiel, Kiel, Germany; 2 Department of Trauma and Orthopedic Surgery, University Medical Center Hamburg-Eppendorf, Hamburg, Germany; 3 Department of Anatomy, Christian-Albrechts- University, Kiel, Germany; 4 Department of Trauma Surgery, Orthopaedics and Sports Traumatology, BG Hospital Hamburg, Hamburg, Germany

**Keywords:** Knee, Tumour, Malignant, Benign, Soft-tissue, Sarcoma, Bone

## Abstract

**Background:**

Due to a lack of routine, there is often uncertainty regarding diagnostics of tumours around the knee joint. This study aimed to provide knowledge about the frequency, distribution and diagnostic algorithm of different bone and soft tissue tumour entities of the knee at a large referral university hospital in Germany.

**Methods:**

Retrospective, longitudinal, single-centre study that reviewed adult patients from 2010 until 2020 with a suspected tumours diagnosis around the knee at a university cancer centre. Inclusion criteria were adults with true bone or soft-tissue tumours in the knee joint and in its adjacent compartments. Suspected diagnosis, histological tumour entity, localization and its surgical treatment by biopsy, resection, osteosynthesis or tumour endoprosthesis were investigated.

**Results:**

A total number of 310 adult patients were included with a mean age of 54.2 ± 18.8 years. In total 160 (51.6%) soft-tissue tumours (69/43.1% benign; 74/46.2% malignant; 17/10.6% intermediate), 92 (29.6%) primary bone tumours (46/50% benign; 39/42.3% malignant; 7/7.6% intermediate), 36 (11.6%) metastases and 22 (7.1%) lymphomas were detected. 171 (55.1%) tumours were classified as malignant. Suspected diagnosis was matched with histology in 74.5% (231/310) of all cases. In 6 cases a primarily suspected benign diagnosis turned out to be malignant. The majority of primary bone tumours was cartilage derived (63.1%;58/92) and located in the distal 2/3 of the femur, whereas intracapsular tumours of the knee joint were rare (13.0%). Soft-tissue tumours were located primarily in the middle third of the thigh (36.8%). The MRI was the diagnostic tool of choice in 98.1% of soft tissue tumours and 82.6% bone tumours.

**Conclusion:**

Awareness is crucial for detecting rare and malignant tumours around the knee, with adipocytic tumours being the most common soft tissue tumour and chondrogenic tumours as the most prevalent malignant bone tumour. Accurate diagnosis of bone tumours necessitates radiographs and frequently an additional MRI scan, while soft tissue tumours require mandatory MRI scans. Incorrectly diagnosing a tumour can have severe consequences, emphasizing the need for histological confirmation in all cases. Additionally, malignant tumours within joint capsules in adults are infrequent.

**Supplementary Information:**

The online version contains supplementary material available at 10.1007/s00402-024-05401-7.

## Introduction

Injuries of the knee joint account for 41% of all sports-related injuries, making it one of the most common musculoskeletal conditions [12]. Elderly people are more likely to have degenerative joint disease, so knee surgery is very common in all age groups. Tumours around the knee joint, on the other hand, are extremely rare accounting for less than 1% of all cancer diagnoses in adults, but the lower extremity is most frequently affected [1, 13]. This often results in uncertainty regarding the diagnosis and treatment of unclear tumours in the knee joint area. In young patients a prolonged bone marrow edema might be suspicious [33]. Older patients age and malignancy suspicion based on magnetic resonance imaging (MRI) reports accelerate the referral to a specialized tumour centre [4]. However, radiologists also infrequently encounter tumour diagnoses, which is reflected in the uncertainty associated with radiological diagnoses. In a study that assessed the quality of primary radiology MRI reports showed frequent deviations from the European Society of Musculoskeletal Radiology (ESSR) guidelines resulting in 25% mismatch with the histopathological diagnosis and 32% misinterpretation of the masses as benign [34]. In only 30% biopsy or referral to a university cancer centre (UCC) was recommended. Swift diagnosis is essentially impossible with the current literature structure, as it is arranged by entities, rendering it unhelpful when the name of the entity is unknown.

Knowledge and diagnosis of these tumours is essential since delayed diagnosis or misinterpretation may lead to limb and life-threatening consequences [17, 29]. Diagnostic delay and improperly performed biopsy must be avoided whenever a tumorous neoplasia is suspected [4]. A European study of over 3000 musculoskeletal tumours including bone (78.1%) and soft-tissue sarcomas (STS; 21.9%) identified 20.7% malignant tumour entities, which were dominantly localized in proximity of the knee joint (femur 26.7%, tibia 20.3%). Along with overall musculoskeletal tumours, in bone and soft-tissue tumours the lower extremity is more frequently affected across all age groups compared to the upper extremity [3, 5]. In a retrospective study of 566 bone tumours the femur was most frequently affected (39.9%) followed by the tibia (17.7%) [3].

Although children and adolescents < 20 years are most commonly affected, malignant tumours have a biphasic distribution with a second peak at the age of 51–55 years [5]. Sport injuries and early osteoarthritis can obscure the presence of tumours in this population [17, 21]. Therefore, special vigilance is necessary throughout any age if a soft-tissue mass or bony irregularity deviate from non-tumour pathologies like baker cyst or meniscal cysts. Descriptive studies so far evaluated single tumour entities or exclusively bone or soft-tissue tumours. Larger studies on specific anatomical localizations and different tumour entities are lacking, which may help in the diagnostic process. This retrospective study examined a total of 367 patients who underwent surgery for a suspected tumour at the University Medical Center Hamburg-Eppendorf in the years 2010–2020 and for whom histopathological findings were available. In addition to frequency and localization, the goal of the study is to illuminate the diagnostic algorithm for tumour diseases of the knee joint in an adult population in northern Europe.

## Materials and methods

Study design: Descriptive cases series with a retrospective, longitudinal data collection from 2010 until 2020 at a university musculoskeletal cancer centre. Inclusion criteria were surgically treated patients with an age > 18 years and any kind of histologically confirmed primary and secondary bone and soft-tissue tumours. Exclusion criteria were space-occupying lesions that were not confirmed as true tumour by histopathology. Indication for surgical treatment was made by the senior surgeon (MP), who has more than ten years of experience in musculoskeletal tumour surgery. Institutional Review Board approval was assigned for this study (WF-071/20).

Suspicious soft-tissue and bone tumours were included up to the adjacent compartments. Anatomical grouping was chosen according to the AO Foundation (Arbeitsgemeinschaft für Osteosynthesefragen) classification system, in which the fracture localisation is defined, using a terminology by creating a 2-element alphanumeric code [20]. The first digit indicates the involved bone (3 femur, 4 tibia). The second digit subdivides the bone into three segments: 1 proximal, 2 diaphyseal, 3 distal. In addition, the code “34” was defined as tumour at the level of the knee joint line or intracapsular of the knee joint.

Following data were evaluated for this study: age, gender, side, imaging findings, tumour location within different anatomical regions in and around the knee, suspected diagnosis and histological analysis including dignity and type of tumour. Cases were included if they could be assigned to bone or soft-tissue tumours according to the World Health Organization (WHO) classification of bone tumours [6, 26].

Statistics: Descriptive statistics were given as mean (range) or percentage. Chi-square test was used to examine the dependency of two categorical variables (groups). *p*-values < 0.05 denote statistical significance. Descriptive statistics are given in mean and standard deviation.

## Results

### Age- and sex-depended tumour distribution

**Fig. 1:**
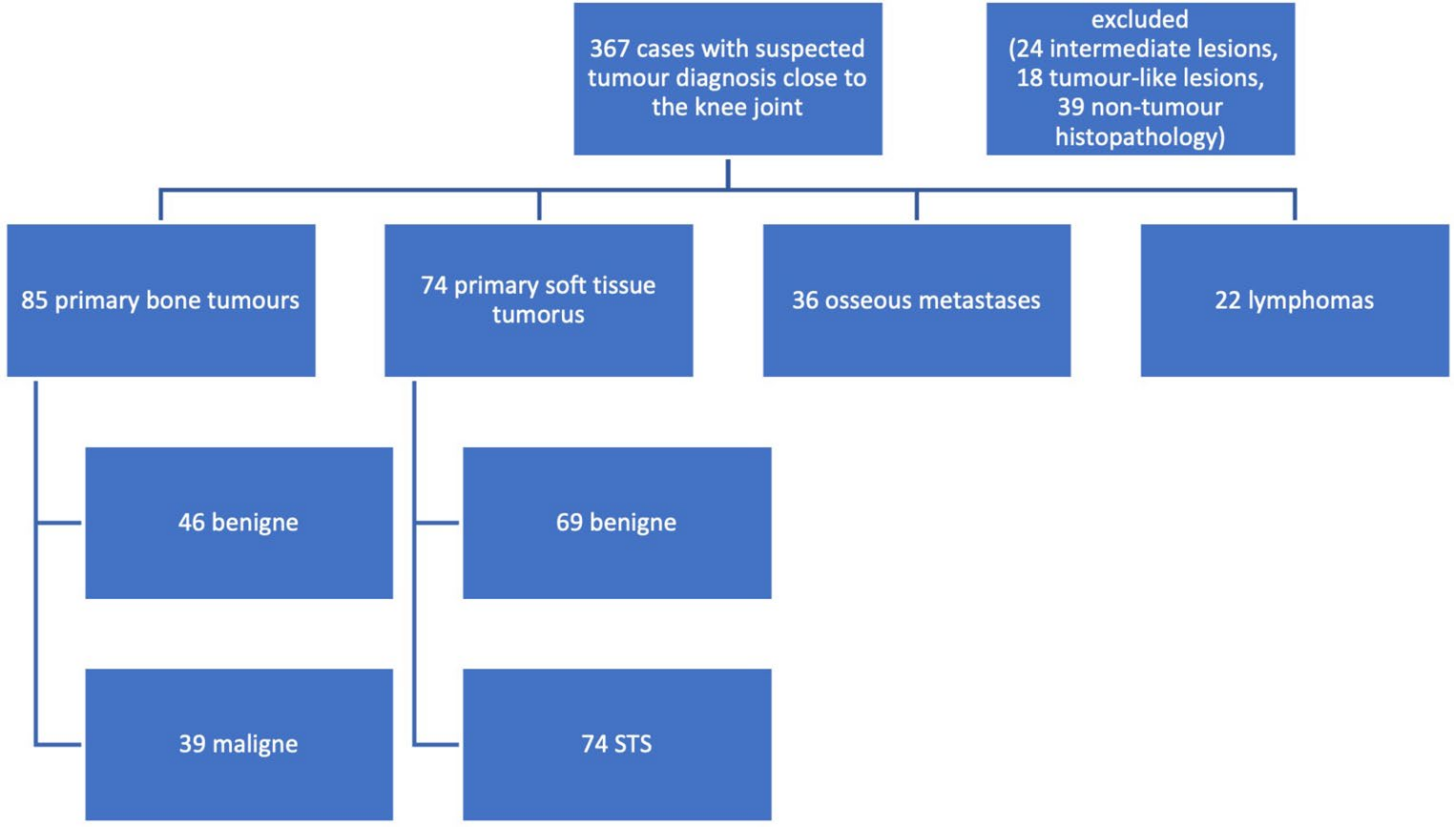
Inclusion criteria of the study. Patients were initially selected with help of the German version (OPS-301) of the ICPM (International Classification of Procedures in Medicine). Tumour entities were classified based on histological reports

A total of 367 patients were screened in the registry (Fig. 1). Patients with intermediate lesions (24), tumour-like lesions (18) and non-tumour diagnosis in the histology (39) were excluded. Therefor the tumour registry accounted a total number of 286 patients with a mean age of 54.3 ± 19.1 years that met the inclusion criteria of being a true tumour. Of these tumours 85 (29.7%) were identified as primary bone tumours, 36 (12.5%) osseous metastases, 22 (7.6%) as lymphomas, 74 (25.8%) as STS and 69 (24.1%) as benign soft tissue tumours. In patients with confirmed tumour diagnosis the suspected diagnosis was confirmed by histology in 70.5% (60/85) of bone tumours and 66.4% (95/143) of soft tissue tumours. In 6 cases a tumour that was primarily considered benign turned out to be malignant (2.1%). The MRI was the diagnostic tool of choice in 98.1% of soft tissue tumours and 82.6% bone tumours.

**Fig. 2:**
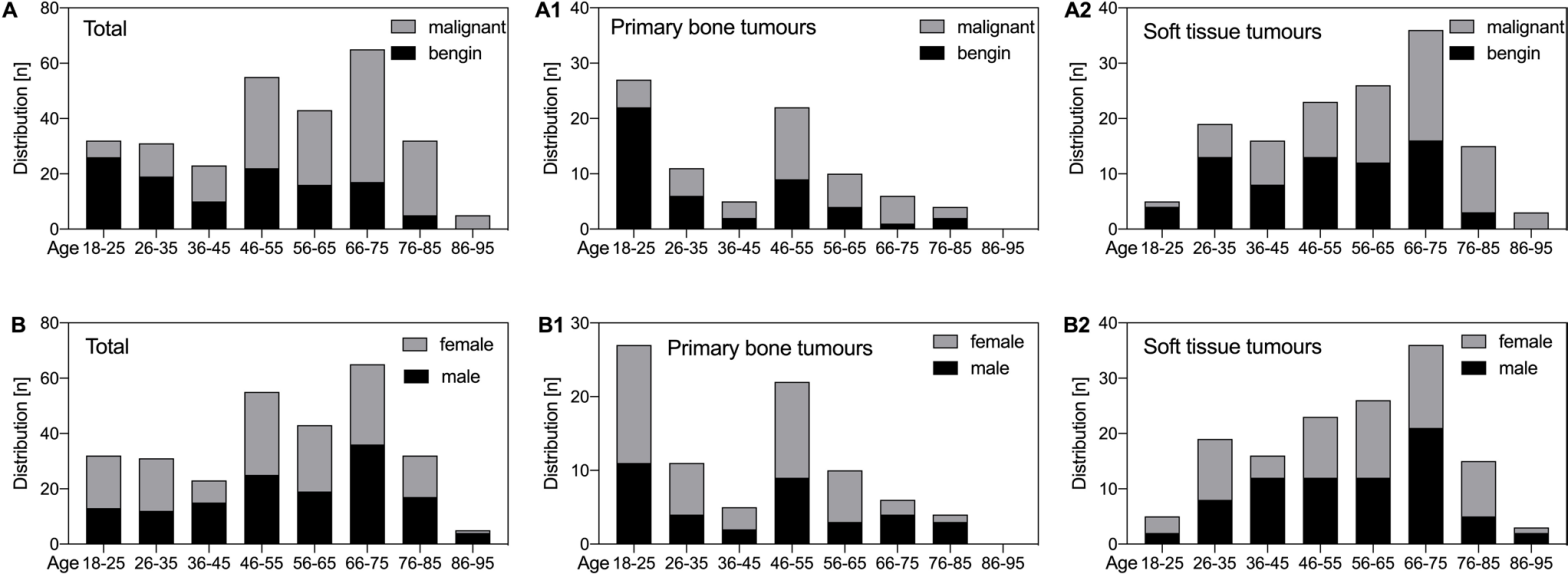
Age-dependent distribution of malignant and benign tumours around the knee in an adult population. (**A**) Distribution in the total population and subdivided by primary bone (**A1**, **B1**) and soft tissue tumours (**A2**, **B2**). The same population was subdivided by male and female sex (**B**)

The age-dependent distribution of primary bone and soft tissue tumours is illustrated in Fig. 2. The average age for all patients with bone tumours was 41.7 ± 18.9 years, for all patients with benign bone tumours 35.19 ± 18.17 years and for all patients with primary malignant bone tumours 49.41 ± 17.09 years (metastases excluded). In soft tissue tumours the average age was 56.77 ± 17.04 years, for all patients with benign soft tissue tumours 51.69 ± 16.07 years and for all patients with STS 61.51 ± 16.64 years. 4 Patients had a bilateral tumour occurrence and a side dominance with respect to the tumour occurrence could not be observed (150 right sided vs. 132 left sided). Osseous metastases were seen in patients with a mean age of 66.19 ± 13.12 years and were most frequently located in the middle 1/3 of the femur (60.5%;23/38). In these, lung-derived tumours (36.1%;13/36) were the most frequent location followed by breast cancer (22.2%;8/36).

### Anatomical tumour location

**Fig. 3:**
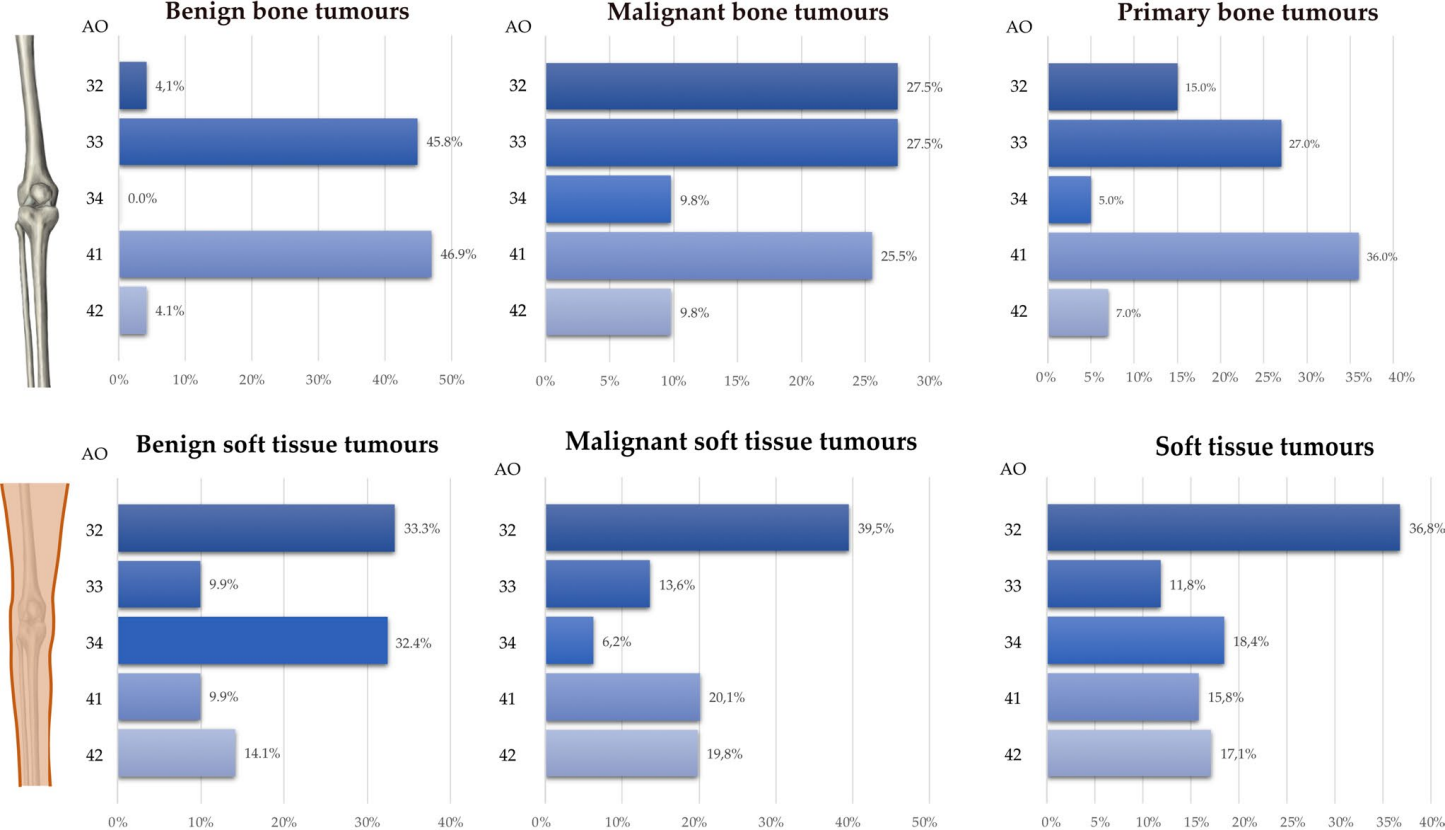
Anatomical localisation of musculoskeletal tumours in adults around the knee. Primary bone (**A**) and soft tissue tumours (**C**) were subdivided in malignant bone (**B**) and soft tissue tumours (**D**). Anatomical locations were categorized according to the AO/OTA classification: 32 femur diaphysis, 31 distal femur, 41 proximal tibia, 42 tibia diaphysis,. Location 34 was defined as a location intracapsular of the knee joint

An overview of the anatomical distribution pattern is given in Fig. 3. The majority of primary bone tumours were identified in the same amount in the distal femur (36%) as well as in the proximal tibia (36%). Among these 61.1% were benign and 38.9% were malignant. Benign bone tumours had a prevalence for the AO41 (46.9%), followed by the AO33 (44.9%) and, lastly, AO32/42 (each approximately 4.0%). Malignant bone tumours were mainly found in the distal two third of the femur (AO32 27.4%, AO33 27.4%), followed by the proximal tibia (AO 41 25.5%). In five cases originating from the distal femur (2 Ewing sarcoma, 3 Chondrosarcoma) and one case originating from the proximal tibia (Parosteal osteosarcoma) the tumour expanded in the knee joint (classified additionally as “AO34”).

Soft tissue tumours were found most frequently (36.8%) at the level of the middle third of the thigh. Regarding this localisation 32 (57.1%;32/56) were being malignant and 25 (44.6%;25/56) were benign. Around the knee joint soft tissue tumours were most frequent distributed at the knee joint line, followed by the proximal third of the tibia and the distal third of the femur. Malignant STS clustered in the middle 1/3 of the thigh. Benign soft tissue tumours were frequently detected in the knee joint and at the level of the femur diaphysis. A significant dependence (*p* < 0.0001) was found between the dignity (malignant vs. benign) and the number of occupied AO segments at the time of tumour diagnosis. Patients with a polysegmental tumour diagnosis were more likely to have a malignant tumour (*p* < 0.05).

### Tumour entities

**Fig. 4:**
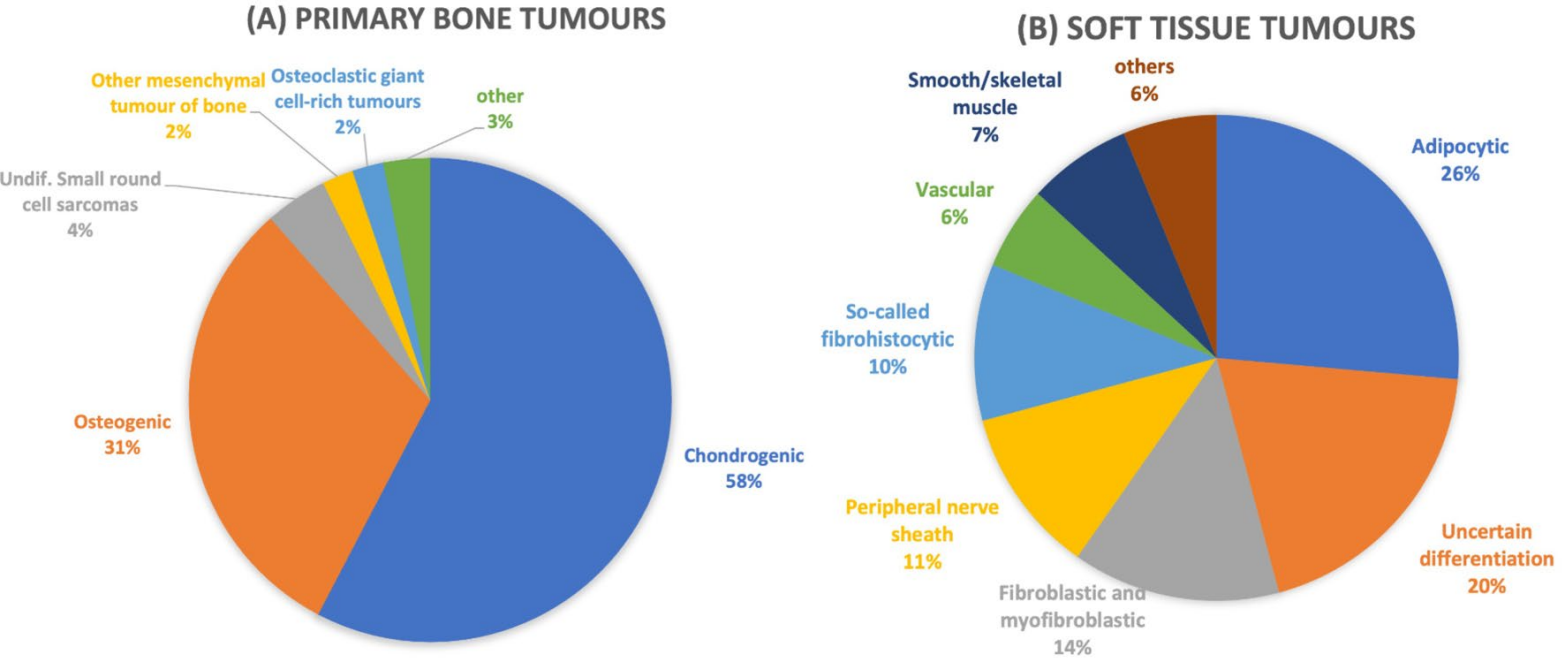
Histopathological analysis of musculoskeletal tumours in adults around the knee. Histopathological analysis of primary bone. (**A**) and soft-tissue tumours (**B**). Tumour lesions were divided into different groups according to WHO classification system

In total 15 different primary bone tumour entities and 38 different soft tissue tumours were identified. The histological origin of primary bone and soft tissue tumours is given in Fig. 4. The following paragraphs briefly list the five most common entities. A detailed overview about the localization, average age of diagnosis, gender distribution and treatment concept of the most frequent primary bone tumours and soft tissue tumours is given in Supplement 1-4.

#### Benign bone tumours

A total of 46 benign bone tumours (16.0%;46/286) were identified with 9 different entities. The top three (Suppl. 1) entities accounted for 84.7% (39/46) of all benign bone tumours. The remaining five entities accounting for 15.2%. Osteochondroma was the most frequent benign bone tumour (52.1%;24/46) followed by enchondroma (19.5%;9/46) and chondroblastoma (13.0%;6/46).

#### Malignant bone tumours

The most common five different malignant tumour entities accounted to 13.6% (39/286) of all tumours. Most frequent entity was the osteosarcoma (43.5%;17/39) followed by chondrosarcoma (38,4%;15/39), Ewing sarcoma (10.2%;4/39), adamantinoma (5.1%;2/39) and osteoclastoma (2.5%;1/39). More detailed data about the three most frequent malignant bone tumours is given in Suppl. 2.

#### Benign soft tissue tumours

A total of 69 (24.1%;69/286) tumours were identified with 19 different entities. The four most frequent entities (Suppl. 3) accounted for 72.4% of all cases. Most two most frequent entities were lipoma (21.7%;15/69) and tenosynovial giant cell tumour (21.7%;15/69). They were followed by schwannoma (20.2%;14/69) and myxoma (8.6%;6/69).

#### Soft tissue sarcoma

Malignant soft tissue tumours were identified in 25.8% (74/286). Most frequent (Suppl.) entity were the tumours of uncertain differentiation (pleomorphic, synovial sarcoma etc.) (28.3%;21/74), followed by liposarcoma (25.6%;19/74), myxo-/fibrosarcoma (21.6%;16/74), angiosarcoma (5.4%;4/74), rhabdomyosarcoma (4.0%;3/74) and leiomyosarcoma (4.0%;3/74).

#### Tumours close to the knee joint

**Fig. 5:**
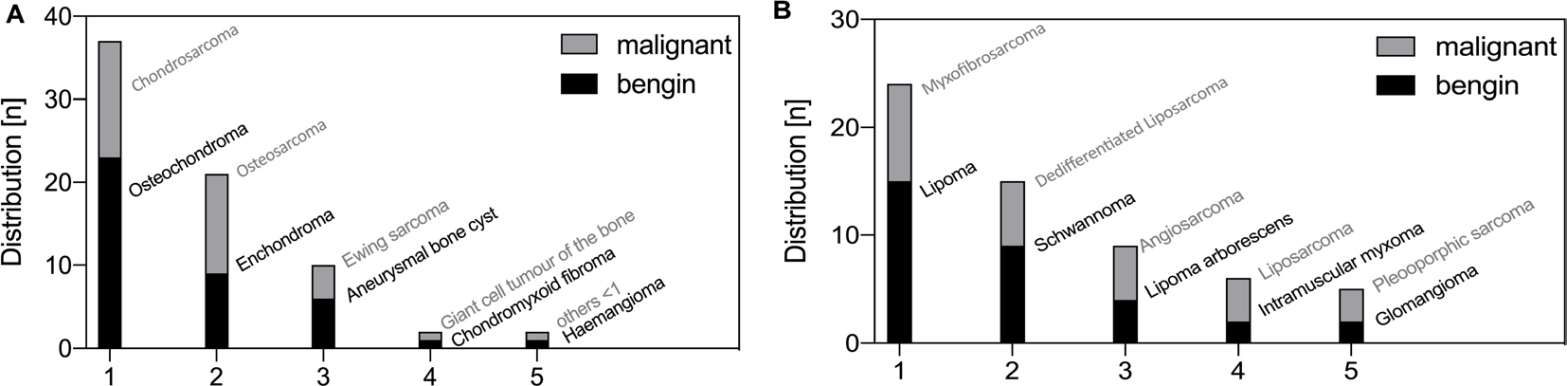
Tumour entities in close proximity to the knee joint (“33 + 34 + 41”): bone tumours (**A**) and soft-tissue tumours (**B**). *pleomorphic rhabdomyosarcoma, leiomyosarcoma, fibrosarcoma, pPNET

In Fig. 5 tumour entities are summarized that can be identified on a conventional x-ray or MRI scan of the knee joint. Intracapsular tumours occurred in 6.9% (20/286). Of these, 80% (16/20) were benign, 20% (4/20) malignant and 2 (< 1%) intermediate lesions. Regarding the 4 malignant tumours we had following histological findings: myxoid liposarcoma, synovial sarcoma, chondrosarcoma and non-hodgkin-lymphoma. Referred to the total number of registered tumours we detected 1.3% (4/286) malignant tumours within the knee joint in this study. Of all intracapsular tumours 19 were treated by resection and 1 needed amputation. Of all tumours in close proximity to the knee joint 61 were malignant and 54 benign.

## Discussion

This study demonstrates the presence of a wide array of different entities around the knee joint, encompassing both soft tissue and bone tumours, with chondrogenic tumours being predominant among bone tumours and lipomatous tumours among soft tissue tumours. In an adult population there were only very few malignant tumours in the knee joint. Malignant tumours are more likely to occur towards the femur diaphysis, but awareness and proper treatment is mandatory as a considerable rate of diagnoses suspected benign turned out malignant in the histology or did not match the suspected diagnosis.

In comparison to other studies about the epidemiology of musculoskeletal tumours, only a few focus on a specific anatomical areas suchas the knee joint [3, 5, 22]. Furthermore, the majority of European studies have concentrated on south and southwestern Europe [3, 11, 22].

Importantly, in this study true intraarticular tumours were rare and commonly benign. Notably, our study results are biased toward malignancies due to the lower likelihood of benign tumors being admitted to a specialized tumor centre, which underlines the observation that few tumours in the knee joint were malignant. Tenosynovial giant cell tumour was most frequent and can be treated by resection and post-operative radiotherapy [30]. In malignant intraarticular tumours, the tumours originated from an extraarticular compartment and progressed the articular knee joint. In proximity to the knee joint, malignant bone tumours were mainly seen in the distal two thirds of the femur and proximal tibia, which lines up with reports in the literature [5, 24]. Most malignant bone tumours were localized in the femur diaphysis and its distal third. A similar pattern was noted for soft-tissue tumours.

In terms of age, in this study there was a second age peak in malignant bone and soft-tissue tumours, while benign bone tumours decreased with age and malignant soft-tissue tumours showed a constant age-related incidence. With respect to bone tumours, various studies have shown that osteosarcoma, Ewing sarcoma and chondrosarcoma are most common malignant bone tumour [10, 24, 27]. Concerning the age distribution, in osteosarcoma there are two peaks occurring in mainly in the second decade and a much smaller one around the 7th decade [10]. In our study we encountered 17 cases of osteosarcomas making it account for 43.5% of all malignant bone tumours. In contrast to the literature, a frequently seen malignant bone tumour in our study was chondrosarcoma accounting for 38.4% of all primary malignant bone tumours. There is a steadily increasing incidence of chondrosarcoma with age also in the United States SEER registries, which may be due to an exposure to oral contraceptives and menopausal hormonal therapy [2]. Summarized, the second age peak in bone tumours must not be overlooked.

Regarding STS the most common histopathological type in Europe have been reported as leiomyosarcoma (19%), liposarcoma (16%), and sarcoma not otherwise specified (NOS, 14%) [25]. However, this seems mainly due to focusing on lower limb STS as there is a large group of uterine leiomycosarcoma in registry data not focused on extremities [27]. In addition, some leiomyosarcoma may be classified as pleiomorphic sarcoma with myogenic differentiation nowadays, which is insufficient to make the true leiomyosarcoma.

With regard to the correct sequence of surgical procedures, taking into account the suspected diagnosis and standard therapy including neoadjuvant treatment methods, no general recommendation can be given based on our study. Whether to perform a first-sage biopsy, excisional biopsy or wide resection is inextricably linked with the suspected tumour diagnosis. Additional parameters like the tumour size, its location relative to the fascia and its radiological appearance must be considered.

For soft tissue tumours, MRI is essential, and ultrasound alone is not adequate for accurate diagnosis (ESMO guidelines) [16]. In calcified lesions and bone tumours conventional radiography in two planes is the first radiological investigation, but there should be a low threshold to carried out a MRI if malignancy cannot be definitely excluded (ESMO guidelines) [28]. MRI of the whole compartment with adjacent joints should be carried out and is currently regarded as the best modality for local staging for tumours of the extremities. Additional CT is not mandatory but may help to evaluate bone stability and may increase sensitivity in rare tumour entities [19]. Noteworthy, a recent study focused on the quality of primary radiology MRI reports and revealed frequent deviations from the European Society of Musculoskeletal Radiology (ESSR) guidelines resulting in 25% mismatch with the histopathological diagnosis and 32% misinterpretation of the masses as benign [34]. In only 30% biopsy or referral to a UCC was recommended.

In soft tissue sarcomas, tumour size and location relative to the fascia are important for assessing malignancy risk [14]. While studies show that size ≥ 5 cm and increased age are main risk factors [7, 15, 32], even smaller lesions can be malignant [8], leading to recommendations for core needle biopsies in tumours ≥ 3 cm [23, 31]. Importantly, in our study there was a relevant discrepancy of the suspected and the histopathologically-confirmed diagnosis, which in 6 cases lead to a malignant tumour diagnosis of a mass primarily considered benign (2.1% of total true tumours). Biopsy-related diagnostic errors including a false diagnosis and incorrect technique have been reported up to 20% in non-trained tumour centres [18]. Therefore, there is a high recommendation to perform a core needle biopsy or open biopsy in a musculoskeletal tumour centre, which achieves a high sensitivity and specificity [9]. For these reasons, ideally, treatment should take place at a center specializing in musculoskeletal tumours.

There are relevant limitations of this study, since this study was designed as a retrospective descriptive case series in a large musculoskeletal tumour centre. This may have led to a preselection bias towards more frequent malignant tumour entities and locally advanced tumour stages recorded in our study and less frequent benign tumour entities. Consequently, there was a high number of tumour-related amputations performed at our centre, which does not necessarily reflect the standard of care especially in bone tumours. In addition, only surgically treated tumours were included, which leads to an underestimation of benign tumour entities. Tumour-like and intermediate lesions were also excluded, which causes a shift towards malignant tumour entities. Hence, the study cohort does not provide a representative sample for soft-tissue and bone tumours, and its interpretation should be considered in conjunction with supplementary literature on individual tumour types.

## Conclusion

Intraarticular tumours of the knee joint are rarely malignant, but care must be taken to diagnose these masses accurately. Age > 45 years, subfascial localisation in STS, tumour size and bone masses of the femur and proximal tibia should arouse attention towards malignancy. Superficial soft-tissue tumours must not be underestimated if the size is ≥ 3 cm. Due to a relevant number of misjudged suspected diagnosis, any treatment should consider previous biopsy and should take place at a centre specializing in musculoskeletal tumours.

## Electronic supplementary material

Below is the link to the electronic supplementary material.

Supplementary Material 1

Supplementary Material 2

Supplementary Material 3

Supplementary Material 4

## Data Availability

The datasets used and/or analysed during the current study are available from the corresponding author on reasonable request.

## References

[1] Amadeo B, Penel N, Coindre J-M et al (2020) Incidence and time trends of sarcoma (2000–2013): results from the French network of cancer registries (FRANCIM). BMC Cancer 20(1):190 32138705 10.1186/s12885-020-6683-0 PMC7059296

[2] Anfinsen KP, Devesa SS, Bray F et al (2011) Age-period-cohort analysis of primary bone cancer incidence rates in the United States (1976–2005). Cancer Epidemiol Biomarkers Prev 20(8):1770–1777 21724855 10.1158/1055-9965.EPI-11-0136

[3] Baena-Ocampo Ldel C, Ramirez-Perez E, Linares-Gonzalez LM, Delgado-Chavez R (2009) Epidemiology of bone tumors in Mexico City: retrospective clinicopathologic study of 566 patients at a referral institution. Ann Diagn Pathol 13(1):16–21 19118777 10.1016/j.anndiagpath.2008.07.005

[4] Ballhause TM, Reiter A, Korthaus A, Frosch KH, Schlickewei CW, Priemel MH (2022) Diagnostic delay in soft tissue tumors: a single-center study of a university cancer center with a focus on health services research. BMC Health Serv Res 22(1):452 35387642 10.1186/s12913-022-07891-w PMC8988367

[5] Bergovec M, Kubat O, Smerdelj M, Seiwerth S, Bonevski A, Orlic D (2015) Epidemiology of musculoskeletal tumors in a national referral orthopedic department. A study of 3482 cases. Cancer Epidemiol 39(3):298–302 25703268 10.1016/j.canep.2015.01.015

[6] Choi JH, Ro JY (2021) The 2020 WHO classification of tumors of soft tissue: selected changes and new entities. Adv Anat Pathol 28(1):44–58 32960834 10.1097/PAP.0000000000000284

[7] Datir A, James SL, Ali K, Lee J, Ahmad M, Saifuddin A (2008) MRI of soft-tissue masses: the relationship between lesion size, depth, and diagnosis. Clin Radiol 63(4):373–378 discussion 379–380 18325355 10.1016/j.crad.2007.08.016

[8] De Marchi A, Pozza S, Charrier L et al (2020) Small Subcutaneous Soft Tissue Tumors (< 5 cm) Can Be Sarcomas and Contrast-Enhanced Ultrasound (CEUS) Is Useful to Identify Potentially Malignant Masses. Int J Environ Res Public Health, 17(23) 10.3390/ijerph17238868 PMC7730454 33260631

[9] Dirks M, Ewerbeck NK, Ballhause TM et al (2023) The diagnostic accuracy of 332 incisional biopsies in patients with malignant tumors in the musculoskeletal system. World J Surg Oncol 21(1):4 36624456 10.1186/s12957-022-02883-w PMC9827702

[10] Duong LM, Richardson LC (2013) Descriptive epidemiology of malignant primary osteosarcoma using population-based registries, United States, 1999–2008. J Registry Manag 40(2):59–64 24002129 PMC4476493

[11] Fabiano S, Contiero P, Barigelletti G et al (2020) Epidemiology of Soft Tissue Sarcoma and Bone Sarcoma inItaly: Analysis of Data from 15 Population-Based Cancer Registries. Sarcoma, 2020:6142613

[12] Gage BE, McIlvain NM, Collins CL, Fields SK, Comstock RD (2012) Epidemiology of 6.6 million knee injuries presenting to United States emergency departments from 1999 through 2008. Acad Emerg Med 19(4):378–385 22506941 10.1111/j.1553-2712.2012.01315.x

[13] García-Ortega DY, Clara-Altamirano MA, Martín-Tellez KS et al (2021) Epidemiological profile of soft tissue sarcomas of the extremities: incidence, histological subtypes, and primary sites. J Orthop 25:70–74 33935434 10.1016/j.jor.2021.03.021 PMC8079324

[14] Gassert FG, Gassert FT, Specht K et al (2021) Soft tissue masses: distribution of entities and rate of malignancy in small lesions. BMC Cancer 21(1):93 33482754 10.1186/s12885-020-07769-2 PMC7825232

[15] Grimer R, Judson I, Peake D, Seddon B (2010) Guidelines for the management of soft tissue sarcomas. Sarcoma, 2010:506182 10.1155/2010/506182 PMC2903951 20634933

[16] Gronchi A, Miah AB, Dei Tos AP et al (2021) Soft tissue and visceral sarcomas: ESMO-EURACAN-GENTURIS clinical practice guidelines for diagnosis, treatment and follow-up(☆). Ann Oncol 32(11):1348–1365 34303806 10.1016/j.annonc.2021.07.006

[17] LiBrizzi CL, Bitzer AM, Kreulen RT, Meyer CF, Morris CD (2022) Sarcoma happens: a reminder for arthroscopic surgeons. Cureus 14(4):e24457 35651443 10.7759/cureus.24457 PMC9132742

[18] Mankin HJ, Mankin CJ, Simon MA (1996) The hazards of the biopsy, revisited. Members of the Musculoskeletal Tumor Society. J Bone Joint Surg Am 78(5):656–663 8642021 10.2106/00004623-199605000-00004

[19] Meyer J, Rolvien T, Reiter A et al (2023) Osteoid osteoma in the bones of the hand: a systematic literature review. Arch Orthop Trauma Surg 143(8):5437–5444 36939892 10.1007/s00402-023-04839-5 PMC10374483

[20] Müller MENS, Koch P (1987) Classification AO Des fractures. Tome I. Les os longs. Springer-

[21] Muscolo DL, Ayerza MA, Makino A, Costa-Paz M, Aponte-Tinao LA (2003) Tumors about the knee misdiagnosed as athletic injuries. J Bone Joint Surg Am 85(7):1209–1214 12851344 10.2106/00004623-200307000-00005

[22] Öztürk R, Arıkan ŞM, Bulut EK, Kekeç AF, Çelebi F, Güngör B (2019) Distribution and evaluation of bone and soft tissue tumors operated in a tertiary care center. Acta Orthop Traumatol Turc 53(3):189–194 30982757 10.1016/j.aott.2019.03.008 PMC6599414

[23] Pavlidis ET, Pavlidis TE (2023) New trends in the surgical management of soft tissue sarcoma: the role of preoperative biopsy. World J Clin Oncol 14(2):89–98 36908679 10.5306/wjco.v14.i2.89 PMC9993143

[24] Picci P (2014) Atlas of Musculoskeletal tumors and Tumorlike lesions. Springer

[25] Saltus CW, Calingaert B, Candrilli S et al (2018) Epidemiology of Adult Soft-Tissue Sarcomas in Germany. Sarcoma, 2018:5671926 10.1155/2018/5671926 PMC5904765 29849478

[26] Sbaraglia M, Bellan E, Dei Tos AP (2021) The 2020 WHO classification of soft tissue tumours: news and perspectives. Pathologica 113(2):70–84 33179614 10.32074/1591-951X-213 PMC8167394

[27] Stiller CA, Trama A, Serraino D et al (2013) Descriptive epidemiology of sarcomas in Europe: report from the RARECARE project. Eur J Cancer 49(3):684–695 23079473 10.1016/j.ejca.2012.09.011

[28] Strauss SJ, Frezza AM, Abecassis N et al (2021) Bone sarcomas: ESMO-EURACAN-GENTURIS-ERN PaedCan Clinical Practice Guideline for diagnosis, treatment and follow-up. Ann Oncol 32(12):1520–1536 34500044 10.1016/j.annonc.2021.08.1995

[29] Thamrongskulsiri N, Limskul D, Tanpowpong T, Kuptniratsaikul S, Itthipanichpong T (2024) Systematic review and meta-analysis of studies comparing cyst wall preservation against cyst wall resection during arthroscopic popliteal cyst decompression. Arch Orthop Trauma Surg 10.1007/s00402-024-05358-7 38700675

[30] Tie K, Wang H, Chen B, Yang X, Chen L (2023) Midterm outcomes of 18 patients with primary intra-articular diffuse tenosynovial giant cell tumor (TGCT) of the knee treated with complete arthroscopic synovectomy and postoperative low-dose radiotherapy at a mean follow-up of 68 months. Arch Orthop Trauma Surg 143(4):2121–2127 35562595 10.1007/s00402-022-04465-7

[31] Tsukushi S, Nishida Y, Yamada Y, Yoshida M, Ishiguro N (2010) CT-guided needle biopsy for musculoskeletal lesions. Arch Orthop Trauma Surg 130(5):699–703 20033699 10.1007/s00402-009-1030-6

[32] Tsukushi S, Nishida Y, Wasa J, Urakawa H, Ishiguro N (2011) Clinicopathological assessment of T1 soft tissue sarcomas. Arch Orthop Trauma Surg 131(5):695–699 21212970 10.1007/s00402-010-1255-4

[33] Villari E, Digennaro V, Panciera A, Ferri R, Benvenuti L, Cesare F (2024) Bone marrow edema of the knee: a narrative review. Arch Orthop Trauma Surg 10.1007/s00402-024-05332-3 PMC11093815 38642163

[34] Weiss S, Korthaus A, Baumann N et al (2021) Musculoskeletal soft-tissue sarcoma: Quality Assessment of initial MRI reports shows frequent deviation from ESSR guidelines. Diagnostics (Basel), 11(4) 10.3390/diagnostics11040695 PMC8069769 33919690

