# Correction to: Not every knee tumour is a ganglion - retrospective analysis of benign and malign tumour entities around the knee

**DOI:** 10.1007/s00402-024-05494-0

**Published:** 2024-08-28

**Authors:** Peter Behrendt, T Grunow, Frosch K-H, M Krause, H Fahlbusch, M Priemel

**Affiliations:** 1 Department of Orthopedic and Trauma Surgery, University Medical Center Schleswig-Holstein, Campus Kiel, Kiel, Germany; 2 Department of Trauma and Orthopedic Surgery, University Medical Center Hamburg-Eppendorf, Hamburg, Germany; 3 Department of Anatomy, Christian-Albrechts- University, Kiel, Germany; 4 Department of Trauma Surgery, Orthopaedics and Sports Traumatology, BG Hospital Hamburg, Hamburg, Germany

**Archives of Orthopaedic and Trauma Surgery**

10.1007/s00402-024-05401-7

In the original version of this article, figure 5 caption was incorrectly given as

Fig. 5 https://www.amazon.de/dp/B0BN7GGM7S?psc=1&ref=ppx_yo2ov_dt_b_product_details: Tumour entities in close proximity to the knee joint (“33 + 34 + 41”): bone tumours (**A**) and soft-tissue tumours (**B**). *pleomorphic rhabdomyosarcoma, leiomyosarcoma, fibrosarcoma, pPNET

but should have been

Fig. 5 Tumour entities in close proximity to the knee joint (“33 + 34 + 41”): bone tumours (**A**) and soft-tissue tumours (**B**). *pleomorphic rhabdomyosarcoma, leiomyosarcoma, fibrosarcoma, pPNET.

The original article has been corrected.

