# Supplementary material for: Not every knee tumour is a ganglion - retrospective analysis of benign and malign tumour entities around the knee
Source: Arch Orthop Trauma Surg. 2024 Jun 21;144(8):3227–34. doi: 10.1007/s00402-024-05401-7 (PMC11417069; doi:10.1007/s00402-024-05401-7)
Supplement: Supplementary file 1 — Supplementary Material 1 [file 402_2024_5401_MOESM1_ESM.docx]

| **Suppl. 1: Benign bone tumours around the knee** | | | | | | | |  |
| --- | --- | --- | --- | --- | --- | --- | --- | --- |
| **Tumour** | **Cases** ‡ | **Age** § | **Age dominance** | **Location(n)** | **Specialty** | **Gender**  **(m/f)** | **Treatment** | **Ref.** |
| Osteochondroma | 24/46  (52.1) | 33±19.2 | 70% between 18-30y | 33x13,41x12, 32x1 | metaphysis (17)  epiphysis (4)  diaphysis (3) | 15 / 9 | 22x EB  2x biopsy 🡒 resection |  |
| - literature based - | 35-50% | | 10-20y; 75% before 20y | 50% around the knee; mainly involved bone segment is the metaphysis. The distal femur is the most common location, followed by proximal tibia and humerus | 30% distal femur, 15-20% proximal tibia | m > f | EB | [9] |
| Enchondroma | 9/46  (19.5) | 45±13.6 | 44% btw 40-50y | 41x5,33x4 | 5xepiphysis  3xmetaphysis  1xdiaphysis | 2/7 | 5x EB  2x biopsy 🡒 resection + compound osteosynthesis  2x EB + compound osteosynthesis |  |
| - literature based - | 10-15% |  | 15-35y | Hand 60%, distal femur 20%, proximal humerus 10%, tibia | Malignant transformation:  Solitary Enchondroma 1%  Olliers disease 25-30%  Mafuccis Syndrome 25-30% | 1/1 | curettage with bone grafting | [5; 6; 11] |
| Chondroblastoma | 6/46  (13.0) | 21±2.5 | 100% btw.18-30 | 3x33,3x41 | Just epiphysis affected | 4/2 | 4x biopsy 🡒 resection + compound osteosynthesis  2x EB + compound osteosynthesis |  |
| - literature based - | 2-5% |  | 19-23y; 80% under 25y | Most common location epiphysis of distal femur and proximal tibia | Local recurrence rate 14-18% | 2/1 | extended intralesional curettage and bone grafting | [1; 3; 4; 8] |
| Osteoid osteoma | 2/46  (4,3) | 32±20.8 | 70% under 30y | 42x2 | ¾ tibia, 50% local recurrence | 2/0 | 1x EB  1x EB + compound osteosynthesis |  |
| - literature based - | 10-14% |  | 10-30y; 70% before 20y | 50% of the osteoid osteomas occur in the lower extremities, especially in the femur and tibia. | Pain usually deteriorates at night and lessens by morning. Characteristically alleviated by salicylates and NSAIDs | 2-3/1 | radiofrequency ablation | [10; 12] |
| Aneurysmatic bone cyst | 1/46  (2,1) | 28 |  | 1x41 | 1xEpi | 1/0 | 1xEB |  |
| - literature based - | 1-2% |  | 10-30y; 75% < 20y | 52% in femur, tibia, humerus, and fibula, most common around the knee | local recurrence rate of  approximately up to 20-25% | 1-1.8/1 | aggressive curettage, with adjuvant treatment (embolisation, cryotherapie, sclerotherapy, radionuclide ablation) and bone grafting | [2; 7] |
| EB excisional biopsy; y years; ‡ n/n total (%); § Mean ± SD | | | | | | | |  |
| **References**  1 Chen W, DiFrancesco LM (2017) Chondroblastoma: An Update. Archives of pathology & laboratory medicine, 141(6):867-871  2 Cottalorda J, Kohler R, Sales de Gauzy J, et al. (2004) Epidemiology of aneurysmal bone cyst in children: a multicenter study and literature review. Journal of pediatric orthopedics. Part B, 13(6):389-394  3 De Salvo S, Pavone V (2022) Benign Bone Tumors: An Overview of What We Know Today. 11(3)  4 Lin PP, Thenappan A, Deavers MT, Lewis VO, Yasko AW (2005) Treatment and prognosis of chondroblastoma. Clinical orthopaedics and related research, 438:103-109  5 Lubahn JD, Bachoura A (2016) Enchondroma of the Hand: Evaluation and Management. The Journal of the American Academy of Orthopaedic Surgeons, 24(9):625-633  6 Mulligan ME (2019) How to Diagnose Enchondroma, Bone Infarct, and Chondrosarcoma. Current problems in diagnostic radiology, 48(3):262-273  7 Rapp TB, Ward JP, Alaia MJ (2012) Aneurysmal bone cyst. The Journal of the American Academy of Orthopaedic Surgeons, 20(4):233-241  8 Springfield DS, Capanna R, Gherlinzoni F, Picci P, Campanacci M (1985) Chondroblastoma. A review of seventy cases. The Journal of bone and joint surgery. American volume, 67(5):748-755  9 Tepelenis K, Papathanakos G, Kitsouli A, et al. (2021) Osteochondromas: An Updated Review of Epidemiology, Pathogenesis, Clinical Presentation, Radiological Features and Treatment Options. In Vivo, 35(2):681-691  10 Tepelenis K, Skandalakis GP, Papathanakos G, et al. (2021) Osteoid Osteoma: An Updated Review of Epidemiology, Pathogenesis, Clinical Presentation, Radiological Features, and Treatment Option. In vivo (Athens, Greece), 35(4):1929-1938  11 Wells ME, Eckhoff MD (2021) Conventional Cartilaginous Tumors: Evaluation and Treatment. 9(5)  12 Zhang Y, Rosenberg AE (2017) Bone-Forming Tumors. Surgical pathology clinics, 10(3):513-535 | | | | | | | |  |
