# Supplementary material for: Not every knee tumour is a ganglion - retrospective analysis of benign and malign tumour entities around the knee
Source: Arch Orthop Trauma Surg. 2024 Jun 21;144(8):3227–34. doi: 10.1007/s00402-024-05401-7 (PMC11417069; doi:10.1007/s00402-024-05401-7)
Supplement: Supplementary file 2 — Supplementary Material 2 [file 402_2024_5401_MOESM2_ESM.docx]

| **Suppl. 2: Malignant bone tumours around the knee** | | | | | | | |  |
| --- | --- | --- | --- | --- | --- | --- | --- | --- |
| **Tumour** | **Cases** ‡ | **Age** § | **Age dominance** | **Location(n)** | **Specialty** | **Gender**  **(m/f)** | **Treatment** | **Ref.** |
| Chondrosarcom | 15/39  (38.4) | 57.6±14.3 | 45-65y | 32x3,33x6,  34x2,41x6,42x1 | 10/26 already metastasized  9/26 two or more segments affected predominantly epiphyse affected  7/26 recurrence | 11/4 | 1x biopsy  2x EB  2x biopsy 🡒 resection  1x biopsy 🡒 resection + compound osteosynthesis  5x biopsy 🡒 resection + endoprothesis  4x biopsy 🡒 resection (amputation) |  |
| - literature based - | 20-25% |  | 40-60y | Femur 41.9% (54% distal), proximal tibia 16.1% | not been shown to be sensitive to chemotherapy or radiation | m>f | Depending on grade from intralesional curettage (Grade 1) to wide surgical excision with multiagent chemotherapy (Grade 3) | [1; 4; 10] |
| Osteosarcoma | 17/39  (43.5) | 45±15.2 |  | 32x10,33x4,  34x1,41x6,42x2, | 7/17  already metastasized  5/17 two or more segments affected | 6/11 | 1xEB  2x biopsy  1x biopsy 🡒 resection  3x biopsy 🡒 resection + compound osteosynthesis  5x biopsy 🡒 resection + endoprothesis  5x biopsy 🡒 resection (amputation) |  |
| - literature based - | 35-50% |  | bimodal distribution:   - 10-14y most common - over 65y | most commonly located in the metaphysis of long bones, especially around the knee in the distal femur (50%) or proximal tibia (19.6%) |  | 1.33/1 | neoadjuvant chemotherapy, wide surgical resection, and adjuvant chemotherapy. | [3; 6] |
| Ewing´s sarcoma | 4/39  (10.2) | 39±20.5 |  | 32x1,33x4,34x,2 | 3/4 2 segments affected  1/4 already metastasized | 4/0 | 2x EB  1x biopsy 🡒 resection  1x biopsy 🡒 resection + endoprosthesis |  |
| - literature based - | 10-15% |  | 10-20y; 80-90% <20 y | 80% in metadiaphysis of long bones: femur>humerus>tibia | incidence is more than seven times higher in whites than in blacks | 1.5/1 | Multimodal therapy with resection, chemotherapy, radiotherapy | [5; 8] |
| Adamantinoma | 2/39  (5.1) | 33±16.3 |  | 2x42 | 1xdiaphysis  1xmetaphysis | 1/1 | 1x biopsy 🡒 resection + compound osteosynthesis  1x biopsy 🡒 resection (amputation) |  |
| - literature based - | 1-2% |  | 20-50y | 97% in long tubular bones, mainly in  diaphysis of the tibia (85-80%) |  | 4-5/1 | extensive resection | [7; 11] |
| Malignant giant cell tumour | 1/39  (2.5) | 75±0 |  | 41 | 1xmetaphysis  recurrence first op. not at a sarcoma center | 0/1 | 1x biopsy 🡒 resection (amputation) |  |
| - literature based - | 2-4% |  | 20-45y | 50% around knee (distal femur or proximal tibia) |  | f>m | extensive resection | [2; 9] |
| EB excisional biopsy; y years; ‡ n/n total (%); § Mean ± SD | | | | | | | |  |
| **References**  1 Alina Maria S, Loredana Gabriela S, Codruta Ileana P, Romulus Fabian T, Roxana F, Andrei M (2012) On the Bone Tumours: Overview, Classification, Incidence, Histopathological Issues, Behavior and Review Using Literature Data. In: M. Enrique Poblet, (ed) Histopathology. IntechOpen, Rijeka, pp Ch. 4  2 Basu Mallick A, Chawla SP (2021) Giant Cell Tumor of Bone: An Update. Current Oncology Reports, 23(5):51  3 Belayneh R, Fourman MS, Bhogal S, Weiss KR (2021) Update on Osteosarcoma. Current Oncology Reports, 23(6):71  4 Cottalorda J, Kohler R, Sales de Gauzy J, et al. (2004) Epidemiology of aneurysmal bone cyst in children: a multicenter study and literature review. Journal of pediatric orthopedics. Part B, 13(6):389-394  5 Eaton BR, Claude L, Indelicato DJ, et al. (2021) Ewing sarcoma. Pediatric Blood & Cancer, 68(S2):e28355  6 Eaton BR, Schwarz R, Vatner R, et al. (2021) Osteosarcoma. Pediatric Blood & Cancer, 68(S2):e28352  7 Kitsoulis P, Charchanti A, Paraskevas G, Marini A, Karatzias G (2007) Adamantinoma. Acta orthopaedica Belgica, 73(4):425-431  8 Riggi N, Suvà ML, Stamenkovic I (2021) Ewing’s Sarcoma. New England Journal of Medicine, 384(2):154-164  9 Singer S, Demetri GD, Baldini EH, Fletcher CD (2000) Management of soft-tissue sarcomas: an overview and update. The Lancet. Oncology, 1:75-85  10 Thorkildsen J, Taksdal I, Bjerkehagen B, et al. (2019) Chondrosarcoma in Norway 1990-2013; an epidemiological and prognostic observational study of a complete national cohort. Acta oncologica (Stockholm, Sweden), 58(3):273-282  11 Varvarousis DN, Skandalakis GP, Barbouti A, et al. (2021) Adamantinoma: An Updated Review. In vivo (Athens, Greece), 35(6):3045-3052 | | | | | | | |  |

S
