# Supplementary material for: Not every knee tumour is a ganglion - retrospective analysis of benign and malign tumour entities around the knee
Source: Arch Orthop Trauma Surg. 2024 Jun 21;144(8):3227–34. doi: 10.1007/s00402-024-05401-7 (PMC11417069; doi:10.1007/s00402-024-05401-7)
Supplement: Supplementary file 3 — Supplementary Material 3 [file 402_2024_5401_MOESM3_ESM.docx]

| **Suppl. 3: Benign soft-tissue tumours around the knee** | | | | | | | |  |
| --- | --- | --- | --- | --- | --- | --- | --- | --- |
| **Tumour** | **Cases** ‡ | **Age** §  **(mean)** | **Age dominance** | **Location(n)** | **Specialty** | **Gender**  **(m/f)** | **Treatment** | **Ref** |
| Lipoma | 15/69  (21.7) | 54±13.1 | 60% btw. 40-60y | 32x9,33x4,34x3,41x1 | 14xSubfascial  1xEpifascial | 6/9 | 15x EB |  |
| - literature based - | 50-60% | | 40-60y | superficial/subcutaneous location is common, most intra-articular  synovial lipomas occur in the knee joint | Size typically plateaus after initial growth | m>f | marginal surgical resection for symptomatic or fastly growing lesions | [3; 6] |
| Schwannoma | 14/86  (20.2) | 52±14.8 | 57,1% btw.30-60y | 32x6,34x4,42x4 |  | 6/8 | 11x EB  3x biopsy 🡒 resection |  |
| - literature based - | 5-10% |  | 30-50y | upper extremity is more often involved than the lower extremity, with a predilection for the median nerve |  | 1/1 | resection | [4; 7] |
| Tenosynovial giant cell tumor | 15/69  (21.7) | 48±19.8 | 30% btw. 20-30y | 34x11,33x3,41x1 | 11/15 intraarticular | 6/9 | 12x EB (3/12 arthroscopy)  3x biopsy 🡒 resection |  |
| - literature based - | 1-5% |  | 30-40y | Diffuse forms are mainly intra-articular, in the knee (75% of cases) |  | 1/1 | arthroscopic or open synovectomy | [2; 5] |
| Intramuscular Hemangioma | 3/69  (4.3) | 49±14.7 |  | 41x2, 32x1 |  | 1/2 | 1x EB  2x biopsy 🡒 resection |  |
| - literature based - | 7% |  | 20-40y | most frequently located in the thigh (36%), followed by the calf (17%) |  | 1/1 | conservative management, systemic corticosteroids, embolization, radiation, sclerotherapy, and surgical excision | [8] |
| Intramuscular Myxoma | 7/69 (8.0) | 57±14.8 | 71% btw.50-75y | 32x4, 41x2, 42x1 | 6 / 7 extensor muscles of legs | 2/5 | 4x EB  3x biopsy 🡒 resection |  |
| - literature based - | 1-5% |  | 40–70y | 50 % of cases commonly occur in the thigh |  | f>m | surgical excision | [1] |
| EB excisional biopsy; y years; ‡ n/n total (%); § Mean ± SD | | | | | | | |  |
| **References**  1 Al Abdulsalam A, Al Safi S, Aldaoud S, Al-Shadidi N, Dhar PM (2022) Intramuscular myxoma of the left thigh: A case report. International journal of surgery case reports, 100:107710  2 Aurégan JC, Klouche S, Bohu Y, Lefèvre N, Herman S, Hardy P (2014) Treatment of pigmented villonodular synovitis of the knee. Arthroscopy : the journal of arthroscopic & related surgery : official publication of the Arthroscopy Association of North America and the International Arthroscopy Association, 30(10):1327-1341  3 Dalal KM, Antonescu CR, Singer S (2008) Diagnosis and management of lipomatous tumors. Journal of surgical oncology, 97(4):298-313  4 Evenski AJ, Stensby JD, Rosas S, Emory CL (2019) Diagnostic Imaging and Management of Common Intra-articular and Peri-articular Soft Tissue Tumors and Tumorlike Conditions of the Knee. The journal of knee surgery, 32(4):322-330  5 Gouin F, Noailles T (2017) Localized and diffuse forms of tenosynovial giant cell tumor (formerly giant cell tumor of the tendon sheath and pigmented villonodular synovitis). Orthopaedics & traumatology, surgery & research : OTSR, 103(1s):S91-s97  6 Hirano K, Deguchi M, Kanamono T (2007) Intra-articular synovial lipoma of the knee joint (located in the lateral recess): a case report and review of the literature. The Knee, 14(1):63-67  7 Magalhães MJS, Pereira DVM, Oliva HNP, et al. (2019) Peripheral Nerve Schwannomas: A Literature Review. Arquivos Brasileiros de Neurocirurgia: Brazilian Neurosurgery, 38(04):308-314  8 Wierzbicki JM, Henderson JH, Scarborough MT, Bush CH, Reith JD, Clugston JR (2013) Intramuscular hemangiomas. Sports health, 5(5):448-454 | | | | | | | |  |
