# Supplementary material for: Not every knee tumour is a ganglion - retrospective analysis of benign and malign tumour entities around the knee
Source: Arch Orthop Trauma Surg. 2024 Jun 21;144(8):3227–34. doi: 10.1007/s00402-024-05401-7 (PMC11417069; doi:10.1007/s00402-024-05401-7)
Supplement: Supplementary file 4 — Supplementary Material 4 [file 402_2024_5401_MOESM4_ESM.docx]

| **Suppl. 4: Malignant Soft-tissue sarcomas** | | | | | | | | | |
| --- | --- | --- | --- | --- | --- | --- | --- | --- | --- |
| **Tumour** | **Cases** ‡ | **Age** § | **Age dominance**  **(% of total)** | **Location** | **Speciality** |  | **Gender (m/f)** | **Treatment** | **Ref.** |
| Liposarcoma | 19/74 (25.6) | 62±16.1 | 57,8% btw. 60-85y | 32x10,33x3,  34x2,41x2,42x3 | 100% subfaszial |  | 8 / 11 | 5x EB  14xbiopsy 🡒 resection(1xamputation) |  |
| - literature based - | 20% |  | 50-60y | more common in lower extremity than upper extremity |  |  | m>f | low grade: marginal resection  intermediate and high grade: extensive resection with adjuvant radiotherapy | [3; 5] |
| Tumours of uncertain dif.  (pleomorph, non-other specified, rundzelliges, spindelzelliges,  synovial Sarkom) | 21/74 (28.3) | 56±17.6 | 61%% btw. 50-80y | 32x12, 33x2,  34x1, 41x4, 42x5 |  |  | 9/12 | 2xEB  13x biopsy 🡒 resection  1x biopsy 🡒 resection + compound-osteosynthesis  1x biopsy 🡒 resection + endoprothesis  4x biopsy 🡒 resection (amputation) |  |
| - literature based -  (Undifferentiated Pleomorphic Sarcoma) | 17.1% |  | 55-80y | extremities most commonly involved location (55%), followed by the trunk (35%), retroperitoneum (9%) |  |  | m>f | surgical excision with radiotherapy and chemotherapy depending on stage | [2; 8] |
| Myxofibrosarcoma | 14/74  (18.9) | 70±11.9 | 71,4% btw. 55-80y | 32x3,33x1,  34x0,41x5,42x6, |  |  | 8/6 | 2xEB  11x biopsy 🡒 resection  1x biopsy 🡒 resection (amputation) |  |
| - literature based - | 5% |  | 50-70 | Lower extremity 52-57%  In muscles and in close vicinity with fascia |  |  | m>f | surgical excision | [7] |
| Pleomorphic Rhabdomyosarcoma | 4/74  (5.4) | 71±17.2 | 100% over 50y | 32x4,34x1 | 100% subfascial |  | 2/2 | 3x biopsy 🡒 resection  1x biopsy 🡒 resection (amputation) |  |
| - literature based - | 1-5% |  | 60-70y | lower extremities |  |  | m>f | wide surgical excision | [1; 6] |
| Angiosarcoma | 4/74  (5.4) | 65±6.8 | 100% over 55y | 32x1  33x1,41x2 |  |  | 3/1 | 4x biopsy 🡒 resection (amputation) |  |
| - literature based - | 2% |  | 60-70y | 10% occur in deep soft tissues  6% of angiosarcomas originate in the bones.  majority (60%) occurs in long bones, with the tibia being the most common site, followed by the femur, humerus and pelvis |  |  | 1/1 (except for cutaneous) | surgical resection when possible, often combined with chemoradiation therapy | [4; 6] |
| **References**  1 Carvalho SD, Pissaloux D, Crombé A, Coindre JM, Le Loarer F (2019) Pleomorphic Sarcomas: The State of the Art. Surgical pathology clinics, 12(1):63-105  2 Chen S, Huang W, Luo P (2019) Undifferentiated Pleomorphic Sarcoma: Long-Term Follow-Up from a Large Institution. 11:10001-10009  3 Dalal KM, Antonescu CR, Singer S (2008) Diagnosis and management of lipomatous tumors. Journal of surgical oncology, 97(4):298-313  4 Gaballah AH, Jensen CT, Palmquist S, et al. (2017) Angiosarcoma: clinical and imaging features from head to toe. The British journal of radiology, 90(1075):20170039  5 Mankin HJ, Mankin KP, Harmon DC (2014) Liposarcoma: a soft tissue tumor with many presentations. Musculoskeletal surgery, 98(3):171-177  6 NOUJAIM J, THWAY K, JONES RL, et al. (2015) Adult Pleomorphic Rhabdomyosarcoma: A Multicentre Retrospective Study. Anticancer Research, 35(11):6213-6217  7 Roland CL, Wang WL, Lazar AJ, Torres KE (2016) Myxofibrosarcoma. Surgical oncology clinics of North America, 25(4):775-788  8 Toro JR, Travis LB, Wu HJ, Zhu K, Fletcher CD, Devesa SS (2006) Incidence patterns of soft tissue sarcomas, regardless of primary site, in the surveillance, epidemiology and end results program, 1978-2001: An analysis of 26,758 cases. International journal of cancer, 119(12):2922-2930 | | | | | | | | | |
